# Nitric Oxide-Releasing Gels in the Context of Antimicrobial Stewardship, Biofilm Management, and Wound-Repair Biology

**DOI:** 10.3390/antibiotics15010054

**Published:** 2026-01-04

**Authors:** Simon J. L. Teskey, Lisa Khoma, Michelle Lorbes, Chris C. Miller

**Affiliations:** SaNOtize Research & Development Corp., Suite 1, 8755 Ash St., Vancouver, BC V6P 6T3, Canada; lkhoma@sanotize.com (L.K.); mlorbes@sanotize.com (M.L.); chris@sanotize.com (C.C.M.)

**Keywords:** general dermatology, medical dermatology, surgery, topical antibiotics, antimicrobial resistance, nitric oxide-releasing gel, biofilm, wound healing, skin infection

## Abstract

Topical antibiotics have long been used for the prevention and treatment of superficial skin and soft tissue infections; however, increasing evidence indicates that their clinical value is undermined by rising antimicrobial resistance, high rates of allergic sensitization, inadequate activity against biofilms, and a lack of wound-healing properties. Agents such as bacitracin, neomycin, polymyxin B, mupirocin, and fusidic acid act through narrow, target-specific mechanisms that facilitate resistance selection and provide limited benefit in chronic or polymicrobial wound environments. Contemporary antimicrobial stewardship frameworks therefore discourage routine use of topical antibiotics and increasingly favor non-antibiotic antiseptics with broad-spectrum activity and low resistance risk, including silver, iodine, polyhexamethylene biguanide, octenidine, and medical-grade honey. These modalities, however, primarily serve to reduce microbial burden and do not directly address the underlying biological impairments that prevent healing. Nitric oxide-releasing gels (NORGs) represent a novel class of topical antimicrobials that combine multi-target bactericidal activity with physiologic pro-healing effects. Nitric oxide exerts potent antimicrobial and antibiofilm effects via oxidative and nitrosative stress, disruption of metabolic pathways, inhibition of DNA replication, and interference with quorum sensing. Simultaneously, nitric oxide enhances angiogenesis, modulates inflammation, improves microvascular perfusion, and promotes fibroblast and keratinocyte function. Preclinical models and early-phase clinical studies demonstrate broad-spectrum efficacy—including activity against multidrug-resistant organisms—with favorable tolerability and minimal risk of resistance development. Although the current evidence base remains preliminary, NORGs offer a promising antimicrobial platform with the potential to reduce reliance on topical antibiotics while simultaneously addressing key barriers to wound healing. Larger randomized controlled trials, direct comparisons with established advanced dressings, and robust pharmacoeconomic evaluations are needed to define their optimal role within stewardship-aligned wound-care practice.

## 1. Introduction

Superficial skin and soft tissue infections (SSTIs) represent a substantial global health burden and remain one of the most common reasons for presentation to primary care, urgent care clinics, and emergency departments worldwide [1,2]. These infections arise from a wide variety of minor injuries, including abrasions, lacerations, superficial burns, and postsurgical incisions, all of which compromise the protective epidermal barrier and permit opportunistic colonization by microorganisms. Their ubiquity is amplified by the rising prevalence of chronic comorbidities such as diabetes mellitus, peripheral vascular disease, and immunosuppression, which impair host defenses and promote wound chronicity [3]. Consequently, SSTIs generate millions of healthcare encounters each year and contribute to significant antimicrobial prescribing and associated stewardship challenges [1,4].

Historically, topical antibiotics such as bacitracin, neomycin, polymyxin B, mupirocin, and fusidic acid have served as the cornerstone of therapy for superficial skin infections. Their widespread use reflects decades of clinical familiarity and the assumption that localized delivery of antimicrobials ensures effective bacterial suppression while minimizing systemic toxicity [2,5,6,7,8,9]. These agents have been incorporated extensively into both prescription and over-the-counter formulations, particularly in triple-antibiotic ointments marketed for domestic wound care and postoperative management [10]. Mupirocin and fusidic acid have additionally acquired essential roles in the treatment of localized impetigo, folliculitis, and nasal or perineal colonization with methicillin-resistant Staphylococcus aureus (MRSA) [11,12,13,14].

However, the scientific and clinical context surrounding topical antibiotics has changed profoundly over the past two decades. Escalating antimicrobial resistance among common SSTI pathogens—including *S. aureus*, MRSA, *Pseudomonas aeruginosa*, and *Enterobacterales*—has eroded the effectiveness of several frontline topical agents [15,16,17,18,19,20]. High-level mupirocin resistance driven by plasmid-mediated mupA and mupB determinants, the global emergence of fusidic acid resistance through fusA and fusB/C/D mechanisms, and the widespread dissemination of aminoglycoside-modifying enzymes have collectively undermined the reliability of these therapies [16,17,18,19]. Parallel to microbial resistance trends, neomycin and bacitracin have become recognized as leading causes of allergic contact dermatitis worldwide, with sensitization rates in dermatologic patch-testing cohorts consistently exceeding 7–13 percent [21,22,23]. These allergic responses can convert otherwise straightforward wounds into recalcitrant inflammatory lesions, further complicating management and reducing therapeutic options.

At the same time, advances in wound-biology research have revealed that many chronic wounds are dominated not by planktonic bacteria susceptible to topical antibiotics but by structured, polymicrobial biofilms that exhibit dramatically heightened tolerance to conventional antimicrobials [24,25,26,27]. Chronic wounds—including diabetic foot ulcers, venous leg ulcers, and pressure injuries—display a multilayered pathophysiology involving persistent inflammation, excessive protease activity, microvascular insufficiency, cellular senescence, oxidative stress, and impaired extracellular matrix remodeling [28,29,30,31]. Biofilms perpetuate inflammatory signaling and immune evasion, creating a self-reinforcing loop that sustains wound chronicity [24,25,26,27]. Conventional topical antibiotics, designed to target actively dividing bacteria, demonstrate limited penetration through biofilm matrices and exert negligible effects on the microenvironmental disturbances that drive impaired healing [2,12,32,33,34].

Reflecting this modern understanding, authoritative guidelines now discourage routine use of topical antibiotics for most acute and chronic wounds. Both the Wound Healing Society and the European Wound Management Association emphasize that chronic wounds are universally colonized and that colonization alone does not warrant antimicrobial therapy [35,36,37]. These guidelines underscore that topical antibiotics provide limited clinical benefit, pose substantial risks of sensitization and resistance selection, and should be used only when clear, localized infection is present [32,35,36]. Instead, non-antibiotic topical antimicrobials—such as silver, iodine, polyhexamethylene biguanide (PHMB), octenidine, and medical-grade honey—are increasingly preferred due to their broad antimicrobial spectra, lower propensity for generating resistance, and greater alignment with antimicrobial stewardship frameworks [38,39,40,41].

Within this shifting therapeutic paradigm, nitric oxide-releasing gels (NORGs) have emerged as an innovative class of topical agents capable of addressing the intersecting microbial, immunologic, and regenerative challenges characteristic of chronic and complex wounds. Nitric oxide (NO) is a ubiquitous endogenous gasotransmitter involved in vascular regulation, innate immune defense against pathogens, modulation of oxidative signaling, angiogenesis, fibroblast activation, and extracellular matrix remodeling [42,43,44,45]. NO exerts rapid, broad-spectrum antimicrobial effects through nitrosative and oxidative reactions that target multiple bacterial processes simultaneously, thereby minimizing the likelihood of classical resistance development [46,47,48]. Advances in NO donor chemistry and biomaterial engineering have enabled the development of hydrogels that release controlled, sustained fluxes of NO directly into the wound bed, thereby combining antimicrobial activity with direct enhancement of tissue repair [49,50,51].

Early experimental and translational studies demonstrate that NORGs eradicate biofilm-embedded bacteria, promote biofilm dispersal through modulation of quorum-sensing pathways, accelerate angiogenesis and re-epithelialization, improve granulation tissue quality, and exhibit a favorable safety profile with minimal risk of sensitization [49,50,51,52,53,54,55]. These attributes position NORGs as promising adjuncts or alternatives to traditional topical antibiotics and align them with contemporary concepts of wound-bed preparation, biofilm-targeted therapy, and antimicrobial stewardship.

The present review provides a comprehensive and deeply integrated analysis of the limitations of classical topical antibiotics, the biological foundations of chronic wound pathology, the molecular and immunologic mechanisms through which nitric oxide supports antimicrobial defense and tissue repair, and the emerging role of nitric oxide-releasing gels in advanced wound care. By synthesizing mechanistic, microbiologic, biomaterial, and clinical evidence, this review contextualizes NORGs within antimicrobial stewardship–driven practice and the broader evolution of topical therapeutics in dermatology and wound management.

## 2. Methods

A structured narrative review methodology was employed to synthesize mechanistic, translational, and clinical evidence regarding conventional topical antibiotics, chronic wound pathophysiology, and nitric oxide-releasing gel technologies. Electronic searches were conducted in PubMed, Scopus, and Web of Science from database inception through November 2025 using combinations of the search terms “topical antibiotics,” “mupirocin,” “fusidic acid,” “bacitracin,” “neomycin,” “polymyxin B,” “biofilm,” “wound infection,” “nitric oxide,” “nitric oxide–releasing gel,” “nitric oxide donors,” “chronic wounds,” “diabetic foot ulcer,” “venous leg ulcer,” “pressure injury,” and “wound healing.” These terms were selected to ensure comprehensive coverage of historical and contemporary literature relating to antimicrobial mechanisms, resistance determinants, wound immunobiology, biofilm behavior, nitric oxide signaling, and biomaterial-based delivery systems [56,57,58].

Sources eligible for inclusion consisted of peer-reviewed mechanistic microbiology studies, molecular and cellular analyses, in vitro and ex vivo biofilm investigations, animal wound-healing models, early-phase human clinical trials, observational human studies, narrative and systematic reviews, and international clinical practice guidelines. Emphasis was placed on foundational and authoritative works elucidating resistance mechanisms such as mupA and mupB in mupirocin resistance, fusA and fusB/C/D in fusidic acid resistance, aminoglycoside-modifying enzymes in neomycin resistance, and plasmid-mediated mcr-1 determinants relevant to polymyxin resistance [59,60,61,62,63]. Similarly, substantial attention was directed toward studies exploring nitric oxide donor chemistry, NO flux measurements, hydrogel-based delivery vehicles, NO-mediated antibiofilm mechanisms, and the influence of NO on angiogenesis, immune modulation, fibroblast proliferation, extracellular matrix deposition, and tissue remodeling [64,65,66,67,68,69].

Key guidelines informing wound-care practice, including those from the Wound Healing Society, the European Wound Management Association, and the Infectious Diseases Society of America, were incorporated given their relevance to antimicrobial stewardship principles, wound-bed preparation, indications for topical therapy, and standards for managing diabetic foot infections and chronic wound colonization versus infection [70,71,72,73]. Reference lists of eligible publications were screened manually to identify additional studies not captured in database searches, with attention to highly cited foundational works and recently published translational investigations.

Data extraction focused on mechanistic themes relevant to modern topical therapy, including bacterial targets of classical antibiotics, molecular determinants of antimicrobial resistance, behaviors and recalcitrance of wound-associated biofilms, host inflammatory and microvascular dysfunction, and nitric oxide-mediated antimicrobial and pro-healing processes. Studies were synthesized narratively to develop an integrated conceptual framework appropriate for expert readership, rather than a quantitative meta-analysis. Priority was given to the convergence of microbiologic, immunologic, and biomaterial engineering findings that directly inform the potential therapeutic role of nitric oxide-releasing gels within contemporary wound management and antimicrobial stewardship paradigms.

## 3. Results

### 3.1. Mechanistic Foundations and Limitations Classical Topical Antibiotics

Classical topical antibiotics have been integrated into clinical practice for decades, supported historically by the assumption that localized, high-concentration antimicrobial delivery provides effective bacterial suppression with minimal systemic exposure. These agents, including bacitracin, neomycin, polymyxin B, mupirocin, and fusidic acid, exert their effects through inhibition of essential bacterial cellular processes. Despite their long-standing use, modern microbiological insights reveal substantial mechanistic, practical, and biological limitations that constrain their effectiveness in contemporary wound care [74,75].

Bacitracin functions by inhibiting the dephosphorylation of bactoprenol pyrophosphate, a lipid carrier required for the translocation of peptidoglycan precursors across the cytoplasmic membrane. This disruption prevents proper cell wall synthesis and leads to bacterial lysis. Its spectrum, however, is largely restricted to Gram-positive organisms such as *Staphylococcus aureus* and *Streptococcus pyogenes*, limiting its utility in polymicrobial or Gram-negative infections commonly encountered in chronic wounds [74]. Neomycin, an aminoglycoside, binds irreversibly to the 30S ribosomal subunit, causing misreading of messenger RNA and inhibition of protein synthesis. Although neomycin possesses a comparatively broader spectrum than bacitracin, including some Gram-negative bacteria, it exhibits poor activity against anaerobes and limited efficacy against important wound pathogens such as *Pseudomonas aeruginosa* [75].

Polymyxin B exerts bactericidal activity by binding to lipid A in the outer membrane of Gram-negative organisms, disrupting membrane integrity and inducing cell death. Its mechanism renders it ineffective against Gram-positive bacteria, thus narrowing its clinical use to wounds in which Gram-negative organisms predominate [76]. Mupirocin, derived from *Pseudomonas fluorescens*, selectively inhibits bacterial isoleucyl-tRNA synthetase, an enzyme essential for the incorporation of isoleucine into nascent proteins. This unique mechanism confers potent activity against *S. aureus*, including methicillin-resistant strains, and *S. pyogenes*, which has contributed to its central role in managing localized staphylococcal infections and MRSA decolonization [77]. Fusidic acid similarly targets bacterial protein synthesis by stabilizing the elongation factor G–ribosome complex and preventing the translocation step of protein elongation, exhibiting primary activity against staphylococci [78].

Although these mechanisms collectively encompass a range of antimicrobial targets, they share several fundamental limitations that diminish their therapeutic utility. Foremost among these is the emergence and rapid dissemination of antimicrobial resistance. Resistance to mupirocin has increased markedly following widespread use for MRSA decolonization, with high-level resistance mediated by plasmid-borne *mupA* and *mupB* genes encoding modified isoleucyl-tRNA synthetases that have reduced affinity for mupirocin [79]. Low-level resistance can arise through point mutations in the native *ileS* gene, further complicating therapeutic decisions [79]. Fusidic acid resistance has proliferated across global regions, driven by mutations in *fusA* and acquisition of resistance genes such as *fusB*, *fusC*, and *fusD*, which encode proteins that protect elongation factor G from drug binding [80]. In many regions, fusidic acid resistance rates have reached thresholds that compromise its clinical effectiveness in impetigo and other superficial infections [80].

Aminoglycoside resistance affecting neomycin derives from several mechanisms, including aminoglycoside-modifying enzymes that acetylate, phosphorylate, or adenylate the drug, ribosomal methylation that reduces drug binding, and active efflux pumps. These mechanisms often reside on mobile genetic elements capable of horizontal transmission among bacteria, raising concerns about cross-resistance to systemically important aminoglycosides [81]. Polymyxin resistance, though initially rare in topical contexts, acquired new urgency following the discovery of plasmid-mediated *mcr-1*, which modifies lipid A and reduces the binding affinity of polymyxins, including polymyxin B used in topical preparations [82]. The mobility of *mcr-1* across *Enterobacterales* underscores the potential collateral risks of indiscriminate topical polymyxin use.

Beyond classical resistance, biofilm-associated tolerance represents a major barrier to the effective use of topical antibiotics in chronic and complex wounds. Biofilms are structured communities of bacteria embedded in an extracellular polymeric matrix that confers up to a thousand-fold reduction in susceptibility to antibiotics compared with planktonic forms [83]. Biofilm-embedded bacteria exhibit heterogeneous metabolic states, including nutrient-limited persister cells that are intrinsically tolerant to drugs targeting cell wall or protein synthesis pathways. The matrix itself impairs penetration of many topical agents, while the coordinated regulation of virulence factors and stress responses via quorum-sensing networks contributes to persistent infection [84]. Chronic wounds, including diabetic foot ulcers, venous leg ulcers, and pressure injuries, frequently exhibit multispecies biofilms composed of *S. aureus*, *P. aeruginosa*, *Enterobacterales*, and anaerobes, rendering them poorly responsive to topical antibiotics designed for acute infections [85].

The limited antimicrobial spectrum of these agents also restricts their utility in complex wounds. Bacitracin and fusidic acid lack activity against Gram-negative species; neomycin displays inconsistent Gram-negative activity and limited anaerobic coverage; and polymyxin B, while effective against certain Gram-negative bacteria, is inactive against Gram-positive organisms such as *S. aureus* [76,77,78]. Chronic wounds often harbor fungi and other nonbacterial organisms, further diminishing the relevance of narrow-spectrum topical antibiotics [85].

In addition to antimicrobial limitations, traditional topical antibiotics carry substantial risks of allergic contact dermatitis (ACD). Neomycin and bacitracin consistently rank among the most common contact allergens in dermatologic practice, with sensitization rates reaching more than ten percent in some patient cohorts [21,22,23]. ACD can cause persistent eczematous inflammation at the wound margins, delay healing, and require discontinuation of the offending agent. Cross-reactivity among aminoglycosides can extend the consequences of sensitization beyond topical therapy, potentially restricting the use of systemically administered aminoglycosides in the future [81].

Finally, these agents lack intrinsic pro-healing properties. They do not improve microvascular perfusion, reduce excessive inflammation, correct protease imbalances, or enhance extracellular matrix deposition, all of which are central to the pathophysiology of chronic wounds [28,29,30,31]. The classical topical antibiotics were developed primarily for short-term treatment of acute, superficial infections and therefore do not address the biological impediments that sustain chronic wound states. As the understanding of wound biology has expanded, these limitations have become increasingly evident, prompting reconsideration of the role of classical topical antibiotics within modern wound-care paradigms and stimulating interest in multifunctional alternatives. A summary of the resistance mechanisms and prevalence for common topical antibiotics is provided in Table 1.

### 3.2. Modern Understanding of Wound Pathophysiology

Advances in wound science over the past three decades have transformed the conceptual framework through which acute and chronic wounds are understood, revealing a complex interplay between microbiology, immunology, vascular biology, matrix remodeling, biofilm behavior, and cellular dysfunction. This modern understanding underscores why many wounds fail to progress through the orderly stages of healing and why classical topical antibiotics, developed primarily for short-duration treatment of acute infections, demonstrate limited benefit in the chronic wound environment [86,87,88].

Acute wound healing unfolds through tightly regulated phases—hemostasis, inflammation, proliferation, and remodeling—in which platelets, neutrophils, macrophages, fibroblasts, endothelial cells, and keratinocytes coordinate to restore tissue integrity. Chronic wounds, by contrast, become arrested in a prolonged inflammatory state characterized by elevated protease activity, destructive oxidative stress, dysfunctional neutrophil and macrophage phenotypes, impaired fibroblast responsiveness, and reduced angiogenic signaling [89,90]. This dysregulated microenvironment creates a biochemical and structural milieu fundamentally resistant to repair.

One hallmark of chronic wounds is persistent inflammation driven by neutrophil extracellular trap formation, excessive cytokine and chemokine production, and impaired macrophage polarization. Normally, macrophages transition from a pro-inflammatory (M1-like) phenotype during early wound defense to a pro-regenerative (M2-like) phenotype that supports extracellular matrix deposition and angiogenesis. In chronic wounds, macrophages fail to undergo this transition, sustaining cycles of tissue injury that contribute to extracellular matrix degradation, increased metalloproteinase activity, and loss of structural integrity in the wound bed [89]. This inflammatory rigidity inhibits the onset of the proliferative phase necessary for healing.

Microvascular dysfunction represents another defining barrier to wound closure. Adequate perfusion is essential for oxygen delivery, nutrient transport, immune cell trafficking, and fibroblast and endothelial cell function. Chronic wounds exhibit impaired vasodilation, microvascular occlusion, endothelial dysfunction, and capillary dropout, particularly in conditions such as diabetic foot ulcers or venous hypertension [91]. Tissue hypoxia exacerbates inflammation, increases susceptibility to infection, and limits the energy-dependent processes required for collagen synthesis, angiogenesis, and epithelial migration [90,91].

Extracellular matrix degradation further contributes to wound chronicity. Excessive matrix metalloproteinase (MMP) activity, accompanied by reduced levels of their endogenous inhibitors, disrupts collagen scaffolding, impairs fibroblast anchorage, and interferes with keratinocyte migration across the wound surface [92]. This biochemical imbalance prevents the formation of stable granulation tissue and obstructs re-epithelialization. Simultaneously, fibroblasts in chronic wounds often exhibit senescence-like phenotypes characterized by diminished proliferative capacity, impaired responsiveness to growth factors, and reduced collagen production, further hindering tissue repair [89,92].

Biofilm presence and behavior impose additional, often underappreciated, barriers to healing. Biofilms develop when bacteria adhere to a wound surface, aggregate, and become embedded within an extracellular polymeric matrix composed of polysaccharides, proteins, lipids, and extracellular DNA. Within this protected environment, microbial populations communicate via quorum-sensing pathways that coordinate virulence expression, regulate nutrient utilization, and modulate immune evasion strategies [93,94,95]. Biofilm-associated bacteria enter metabolically altered states that confer profound tolerance to both antibiotics and host immune responses, enabling them to persist despite apparently appropriate antimicrobial regimens. In chronic wounds, biofilms are not incidental but foundational components of the pathogenic architecture, with prevalence estimates exceeding eighty percent in some clinical cohorts [93].

The consequences of biofilm structure extend beyond antimicrobial tolerance. Biofilms perpetuate inflammation by continuously stimulating neutrophil recruitment, promoting oxidative bursts, and triggering cycles of tissue destruction. Detached biofilm fragments can seed new sites within the wound bed, expanding the microbial footprint and contributing to spatially heterogeneous pockets of infection [94]. Because topical antibiotics diffuse poorly into biofilm matrices and lack activity against nondividing or persister cells, they are frequently rendered ineffective in these environments [32,83]. The complex architecture of chronic wounds therefore limits the utility of antibiotics designed for planktonic bacteria.

Endogenous growth-factor dysregulation also plays a central role in wound stagnation. Chronic wounds exhibit reduced levels of vascular endothelial growth factor (VEGF), platelet-derived growth factor (PDGF), and transforming growth factor-beta (TGF-β), all of which are essential for fibroblast recruitment, endothelial cell proliferation, angiogenesis, and collagen deposition. At the same time, protease-rich environments degrade these growth factors shortly after they are produced, preventing effective signaling [92]. This disruption undermines the proliferative and remodeling phases required for durable closure.

Furthermore, chronic wounds frequently contain mixed populations of aerobic and anaerobic bacteria, fungi, and sometimes viral elements. The polymicrobial nature of these wounds contributes to metabolic cross-feeding, cooperative virulence, and ecological stability within the biofilm community, allowing for more effective resistance to antimicrobial pressures [95]. Many of these organisms, including *P. aeruginosa*, *S. aureus*, *Enterobacterales*, and anaerobes, exhibit synergistic interactions that enhance persistence and impair healing.

Compounding these biological challenges, systemic conditions such as diabetes mellitus exacerbate chronic wound pathology by impairing leukocyte chemotaxis, reducing nitric oxide bioavailability, compromising endothelial responsiveness, and promoting advanced glycation end-product formation that stiffens tissues and disrupts cell–matrix interactions [91]. Peripheral arterial disease, venous insufficiency, autoimmune conditions, and neuropathy similarly contribute to a pathological microenvironment hostile to wound repair.

Collectively, this modern pathophysiological framework reveals why antimicrobial monotherapy—particularly with narrow-spectrum topical antibiotics—is insufficient for managing chronic wounds. Addressing biofilm recalcitrance, inflammatory dysregulation, extracellular matrix degradation, microvascular impairment, and cellular dysfunction requires therapeutic modalities that target multiple biological domains simultaneously. These insights have catalyzed the shift toward antimicrobial stewardship–driven wound care and the exploration of advanced agents, such as nitric oxide-releasing gels, that exert multi-domain antimicrobial and pro-healing effects. Understanding the complexities of chronic wound biology thus provides essential context for evaluating both the limitations of classical topical antibiotics and the promise of emerging therapeutic approaches.

### 3.3. Nitric Oxide Biology and Mechanisms Relevant to Wound Healing

Nitric oxide (NO) is a small, highly diffusible, redox-active gasotransmitter that exerts wide-ranging effects on vascular homeostasis, immune regulation, antimicrobial defense, cellular signaling, and tissue repair. Since its discovery as endothelium-derived relaxing factor and subsequent recognition as a ubiquitous mediator of cell communication, NO has been increasingly understood as indispensable to cutaneous wound healing. Dysregulation of NO production is now recognized as a hallmark of chronic wounds, while exogenous NO delivery has emerged as a means of restoring microenvironmental balance in impaired tissues [96,97,98].

Endogenous NO synthesis is catalyzed by three nitric oxide synthase (NOS) isoforms—endothelial NOS (eNOS), neuronal NOS (nNOS), and inducible NOS (iNOS)—each of which plays a distinct role in skin physiology and repair. eNOS produces low levels of NO in a constitutive, calcium-dependent manner, regulating basal vascular tone, microcirculatory flow, and endothelial cell function [99]. nNOS, expressed in peripheral neurons and certain cutaneous cells, contributes to neurogenic inflammation and neuromodulation within the skin. By contrast, iNOS is transcriptionally upregulated during inflammation in macrophages, neutrophils, keratinocytes, and fibroblasts, generating high-output, calcium-independent NO as part of the innate immune response [100]. This surge of iNOS-derived NO is particularly important for pathogen control, as it enables potent oxidative and nitrosative microbial killing.

NO exerts antimicrobial effects across multiple molecular targets simultaneously, distinguishing it from conventional antibiotics that typically act at single, highly specific sites. NO reacts with oxygen and superoxide to form reactive nitrogen species such as peroxynitrite and dinitrogen trioxide, which damage bacterial membranes, disrupt respiratory-chain enzymes, oxidize protein thiols, cause base deamination, and induce DNA strand breaks [101,102,103]. These mechanisms collectively impair fundamental microbial processes including energy metabolism, structural integrity, and genetic stability. Because these processes are diverse and redundantly targeted, the likelihood of bacteria developing classical resistance to NO through single-gene mutations is exceedingly low [104].

In addition to its antimicrobial properties, NO plays a central role in regulating inflammation, which is critical for the orderly progression of wound healing. NO modulates leukocyte adhesion to the endothelium, reduces excessive neutrophil infiltration, and influences macrophage polarization. By facilitating the transition from pro-inflammatory (M1-like) macrophages to pro-regenerative (M2-like) phenotypes, NO helps resolve inflammation and encourages progression into the proliferative phase of healing [105]. This immunoregulatory capacity is particularly relevant in chronic wounds, where macrophage polarization is often disrupted, leading to persistent inflammation and tissue degradation [89,92].

NO also influences multiple aspects of the proliferative phase, including angiogenesis, fibroblast activity, and extracellular matrix deposition. NO stimulates endothelial cell migration and proliferation, in part through activation of soluble guanylate cyclase (sGC) and subsequent elevation of cyclic guanosine monophosphate (cGMP) levels. The sGC–cGMP–protein kinase G signaling pathway promotes vasodilation, increases capillary perfusion, and drives new vessel formation—a process essential for restoring oxygenation and metabolic support to healing tissues [88]. In fibroblasts, NO enhances proliferation, collagen synthesis, and cytoskeletal organization, thereby strengthening granulation tissue and improving tensile properties of healed wounds [92]. These effects help counteract the fibroblast senescence and growth-factor deficiencies characteristic of chronic wounds.

Keratinocyte migration, essential for re-epithelialization, is similarly regulated by NO. Low to moderate NO concentrations promote keratinocyte motility and directional migration across the wound bed, while excessive NO may hinder keratinocyte differentiation. This concentration-dependent duality is characteristic of NO biology and highlights the importance of controlled delivery for therapeutic purposes [106]. Chronic wounds, which often exhibit insufficient endogenous NO production due to microvascular disease, oxidative stress, and endothelial dysfunction, consequently, fail to support efficient epithelial resurfacing [91,92,106].

Beyond cellular-level effects, NO also exerts profound influence on tissue perfusion and microvascular architecture. NO-mediated vasodilation enhances oxygen diffusion and nutrient transport into hypoxic wound beds, mitigating conditions that foster biofilm persistence and protease-driven matrix breakdown. Improved perfusion is closely linked to more efficient immune-cell trafficking, facilitating bacterial clearance and bolstering host defenses [88]. These microvascular benefits are especially critical in diabetic foot ulcers, pressure injuries, and ischemic wounds, in which baseline perfusion is severely compromised [91].

NO also contributes to antioxidant defense and modulation of redox balance in the wound environment. While NO can combine with superoxide to form peroxynitrite, a potent oxidant, NO also acts indirectly to reduce oxidative stress by inhibiting NADPH oxidase activity and modulating mitochondrial electron transport. In physiologic concentrations, NO therefore helps temper excessive reactive oxygen species accumulation, thereby reducing oxidative injury to keratinocytes, fibroblasts, and endothelial cells [107]. Chronic wounds, which typically exhibit high oxidative stress and impaired antioxidant responses, can benefit from restoration of balanced redox signaling [90].

Taken together, these molecular actions illustrate why NO is fundamental to host defense and tissue repair, and why exogenous NO supplementation may correct key deficiencies in chronic wound biology. Its antimicrobial, immunomodulatory, angiogenic, perfusion-enhancing, and matrix-stabilizing properties position NO as a uniquely multifunctional mediator capable of addressing the intertwined pathological processes characteristic of impaired wounds. These functional roles are summarized in Table 2. The therapeutic implications of these mechanistic insights form the foundation for the development of nitric oxide-releasing gels and other NO-delivery platforms designed to restore physiological NO signaling and support the microenvironmental shifts required for effective wound healing.

### 3.4. Nitric Oxide-Releasing Gels: Chemistry, Delivery Systems, and Biological Advantages

Nitric oxide-releasing gels (NORGs) represent a rapidly advancing class of topical therapeutics designed to harness and deliver NO in controlled, sustained, and clinically meaningful concentrations. These technologies reflect substantial progress in biomaterial engineering, donor chemistry, and wound-healing science, combining broad-spectrum antimicrobial action with host-directed biological benefits. Their emergence reflects decades of effort to overcome the inherent challenges posed by NO’s short half-life, gaseous state, concentration-dependent bioactivity, and susceptibility to rapid oxidation in biological environments [108,109,110].

The chemistry underlying NORGs typically involves the incorporation of NO donors—compounds capable of releasing NO under physiological conditions—within biocompatible hydrogel matrices. Several donor classes have been adapted for topical use. Acidified nitrite systems, modeled after the antimicrobial effects of acidified nitrite in gastric and mucosal environments, release NO via proton-mediated reduction of nitrite ions, generating bursts of NO that can be modulated by pH and buffer composition [111]. Diazeniumdiolates are another donor class characterized by predictable, stoichiometric release of NO upon exposure to aqueous environments. Their decomposition kinetics vary according to structural modifications, enabling tuning of release half-lives from seconds to hours [112]. S-nitrosothiols (RSNOs), naturally present in plasma and tissues, release NO via homolytic cleavage of the S–N bond, often triggered by heat, light, trace metals, or reducing agents. Synthetic RSNO-based donors incorporated into hydrogels allow for sustained, low-level NO release resembling physiological signaling [113].

More recently, nitrite-containing silica or glass matrices have been developed to generate NO upon contact with aqueous fluids or through chemical reduction pathways mediated by endogenous components of the wound bed. These glass or xerogel platforms offer enhanced stability, the ability to control release kinetics through doping and structural modification, and high resistance to thermal degradation [114]. Collectively, these donor systems enable fine modulation of NO dose, flux, and duration, providing distinct therapeutic profiles relevant to wound type, microbial burden, and host physiology.

Hydrogels, as delivery vehicles, offer several advantages that make them particularly suitable for topical NO therapy. Their high water content and porous structure facilitate gas diffusion while maintaining moist wound-healing environments known to support cellular migration, granulation tissue development, and angiogenesis [115]. Hydrogels also conform to irregular wound geometries, adhere gently to wound surfaces without causing trauma upon removal, and can incorporate multiple functional components, including NO donors, antioxidants, pH modulators, or additional bioactive molecules [115,116]. Their rheological properties enable even distribution of NO across the wound surface, while their swelling behavior can modulate NO diffusion and sustain release over hours to days [116].

A central advantage of NORGs is their ability to deliver NO in precise, therapeutic fluxes that balance antimicrobial potency with pro-healing effects. High concentrations of NO can be cytotoxic, whereas moderate concentrations stimulate endothelial, fibroblast, and keratinocyte function. By embedding donors within hydrogels, NORGs achieve stable release kinetics, minimizing the risk of delivering excessively high NO concentrations that could cause oxidative injury [108,109,110,113]. This controlled delivery mimics physiologic NO signaling and has been shown to enhance angiogenesis and collagen deposition in animal wound models [117].

Evidence from preclinical studies consistently demonstrates the broad antimicrobial efficacy of NORGs. In vitro investigations show that NO released from hydrogels rapidly kills Gram-positive and Gram-negative bacteria, including multidrug-resistant strains such as MRSA, *Pseudomonas aeruginosa*, vancomycin-resistant enterococci, and carbapenem-resistant *Enterobacterales* [117,118,119]. NO’s antibiofilm capabilities are particularly notable. NO stimulates biofilm dispersal by interfering with cyclic-di-GMP signaling pathways, a key regulator of the transition between planktonic and biofilm states [120]. This disruption enhances susceptibility of biofilm-associated bacteria to antimicrobial agents and host immune defenses, addressing a major challenge in chronic wound management.

Animal studies extend these microbiologic findings by demonstrating consistent improvements in wound closure, granulation tissue quality, epithelial thickness, and tensile strength following treatment with NO-releasing gels. Rodent models of infected and noninfected wounds treated with NORGs exhibit accelerated re-epithelialization, enhanced angiogenesis, and significant reductions in bacterial burden compared with wounds treated with standard dressings or antibiotic ointments [117,118,119,120,121]. These results highlight the dual antimicrobial and regenerative potential of NO-based therapies.

NORGs also exhibit favorable safety profiles in preclinical and early-phase human studies. Systemic absorption of NO or its oxidation products such as nitrate and nitrite is minimal following topical application, and no significant effects on systemic hemodynamics have been reported [122]. Local tolerability is generally excellent, with only mild, transient irritation observed in some cases. Importantly, no cases of allergic contact dermatitis attributable to NORGs have been documented, distinguishing them from neomycin- or bacitracin-based ointments, which demonstrate high sensitization rates [21,22,23]. The absence of sensitization may reflect both NO’s endogenous nature and the inert character of many hydrogel polymers used in NORG formulations [122].

Stability and manufacturing considerations remain active areas of investigation. NO donors can degrade during storage, reducing therapeutic potency if not adequately stabilized. Advances in xerogel encapsulation, donor surface modification, and polymer chemistry have improved shelf stability, mitigated premature NO release, and enhanced reproducibility of donor activation under clinical conditions [114,123]. Standardization of NO delivery is another priority, as flux measurements can vary substantially depending on donor type, gel thickness, hydration state, and wound exudate composition [108,124]. Current efforts aim to develop practical standards for NO flux characterization to ensure consistency across formulations.

Despite these challenges, the biological advantages of NORGs remain compelling. Their multi-target microbial killing, low resistance potential, antibiofilm efficacy, pro-angiogenic and pro-reparative signaling, and avoidance of sensitization position them as promising next-generation agents within antimicrobial stewardship frameworks. These properties directly address limitations of conventional topical antibiotics and align with contemporary concepts of wound-bed preparation and biofilm-targeted management. As advancements continue in donor chemistry and hydrogel engineering, the translational potential of NORGs is expected to expand further across diverse wound types.

### 3.5. Comparative Limitations of Topical Antibiotics Within Antimicrobial Stewardship Frameworks

Antimicrobial stewardship (AMS) has become a central guiding principle in modern wound care, driven by the global imperative to curb antimicrobial resistance and to rationalize the use of all antibiotic classes, including topical formulations. Historically, topical antibiotics were assumed to pose minimal risk to stewardship because of their localized activity and limited systemic absorption. However, accumulating evidence demonstrates that topical agents contribute meaningfully to the selection and dissemination of resistance determinants, and that their widespread, often prophylactic use can undermine both patient-level and public health outcomes [125,126]. Within AMS frameworks, the limitations of classical topical antibiotics have therefore become increasingly apparent, prompting re-evaluation of their role in acute and chronic wound management.

One of the primary stewardship concerns stems from escalating resistance associated with commonly used topical antibiotics. Mupirocin, once considered a cornerstone of MRSA decolonization efforts, now faces rising rates of high-level resistance mediated by plasmid-borne *mupA* and *mupB* genes, which encode modified isoleucyl-tRNA synthetases with markedly reduced affinity for the drug [79]. In healthcare environments where mupirocin is used widely for decolonization, resistance prevalence can exceed fifteen to twenty percent, reducing its efficacy and undermining infection-control strategies aimed at preventing MRSA outbreaks [125]. Similarly, fusidic acid resistance has expanded globally, driven by mutations in *fusA* and acquisition of *fusB/C/D* genes that protect elongation factor G from drug inhibition [80]. These resistance determinants not only compromise topical efficacy but can also confer cross-resistance affecting systemic treatment options for invasive staphylococcal infections [126].

Another major stewardship issue involves the contribution of topical antibiotics to the selection of multidrug-resistant organisms (MDROs) in polymicrobial wound environments. Repeated exposure to subtherapeutic antibiotic concentrations—common in biofilm-laden chronic wounds where drug penetration is limited—creates selective pressure that favors resistant subpopulations [127]. Biofilms enable localized regions of high bacterial density, slow growth, and enhanced horizontal gene transfer, all of which accelerate the emergence of resistance within the wound ecosystem [93,94,95,127]. Chronic wounds often serve as reservoirs of antibiotic-resistant bacteria that can disseminate to other body sites or transmit to community and healthcare contacts, raising the broader public health stakes of inappropriate topical antibiotic use [128].

Beyond resistance selection, the narrow antimicrobial spectra of traditional topical antibiotics conflict with the polymicrobial and biofilm-dominant nature of chronic wounds. Agents such as bacitracin and fusidic acid lack Gram-negative coverage, while neomycin demonstrates inconsistent activity against anaerobes and limited effect on important wound pathogens such as Pseudomonas aeruginosa [74,75,76,77,78]. In chronic wounds where bacterial communities consist of aerobes, anaerobes, and fungi, narrow-spectrum antibiotics may suppress susceptible organisms while leaving resistant or intrinsically tolerant pathogens unopposed, potentially altering microbial ecology in ways that worsen wound status or impede healing [129].

Stewardship frameworks also emphasize the risks associated with allergic contact dermatitis (ACD), which is notably prevalent with neomycin and bacitracin. Sensitization rates in patch-tested populations frequently exceed ten percent, and the risk increases with repeated or prolonged application [21,22,23]. ACD not only delays healing but can mimic or exacerbate wound infection, leading to diagnostic confusion and unnecessary antibiotic escalation. From an AMS perspective, agents with high sensitization potential and limited clinical benefit present disproportionate risks relative to their therapeutic value [128,129].

Contemporary guidelines from the Wound Healing Society, European Wound Management Association, and Infectious Diseases Society of America reflect these concerns by recommending against the routine use of topical antibiotics for uncomplicated acute wounds, colonized chronic wounds, and most cases of superficial inflammation without overt infection [35,36,37,70,71,72,73]. These guidelines uniformly stress that colonization alone does not warrant antimicrobial treatment, and that overuse of topical antibiotics can inadvertently select for resistant organisms while performing poorly in biofilm-rich wound beds. Instead, they encourage the use of antimicrobial dressings, debridement, moisture management, offloading, and optimization of systemic factors—interventions with stronger evidence bases for improving healing trajectories [130].

The limited capacity of topical antibiotics to disrupt mature biofilms further undermines their stewardship value. Biofilms exhibit up to 1000-fold increased tolerance to antibiotics, and persister cells within biofilms can survive even high concentrations of bactericidal agents [83,84]. Topical antibiotics are generally unable to eradicate biofilms because they target pathways active in metabolically robust planktonic bacteria, not the quiescent or heterogeneously active populations within biofilms [32,83,127]. Consequently, treatment with topical antibiotics may produce partial suppression of planktonic cells while leaving biofilms intact, creating cycles of recurrent infection and inflammation that foster chronicity and drive excessive healthcare use [94,95,130].

AMS frameworks prioritize broad-spectrum, low-resistance-risk antimicrobials and non-antibiotic agents capable of addressing biofilm-associated pathology. Silver, iodine, polyhexamethylene biguanide (PHMB), octenidine, and medical-grade honey have accordingly become preferred topical antimicrobials because of their multifaceted mechanisms of action, low rates of acquired resistance, and broad applicability across contaminated or critically colonized wounds [38,39,40,41,130]. These agents act through multimodal pathways that reduce the likelihood of target-specific resistance and exhibit variable antibiofilm activity, better aligning with modern wound microbiology than narrow-spectrum topical antibiotics [38,129].

In this stewardship context, classical topical antibiotics now occupy a limited therapeutic niche. They retain value for targeted treatment of specific superficial infections such as localized impetigo, limited folliculitis, or traumatic wounds with clear signs of infection caused by susceptible organisms [11,12,13,14,77]. However, their routine use in prophylaxis or uninfected wounds lacks evidence, presents avoidable risks, and contradicts the principles of responsible antimicrobial use [128,129,130]. As wound care shifts toward strategies emphasizing biofilm control, host-modulating therapies, and broad-spectrum non-antibiotic antimicrobial dressings, the rationale for habitual application of classical topical antibiotics continues to diminish.

Within this evolving paradigm, emerging topical agents such as nitric oxide-releasing gels offer potential alignment with stewardship principles due to their multi-target antimicrobial actions, inherent antibiofilm effects, low resistance potential, and concurrent pro-healing properties. Their role, however, must be evaluated rigorously within AMS frameworks, ensuring that adoption is grounded in evidence and that these agents complement—not replace—essential systemic antibiotic therapies for deep or systemic infections. Understanding the comparative limitations of traditional topical antibiotics is therefore essential for situating novel modalities within modern, stewardship-oriented wound-management pathways.

### 3.6. Comparative Evaluations of NORGs and Advanced Non-Antibiotic Wound Dressings

The evolution of wound-care practice toward antimicrobial stewardship has fostered a strong shift away from classical topical antibiotics and toward advanced non-antibiotic antimicrobials, including silver, iodine, polyhexamethylene biguanide (PHMB), octenidine, and medical-grade honey. These modalities are favored because they offer broad-spectrum antimicrobial activity, reduced risk of resistance selection, antibiofilm effects, and favorable alignment with guideline-based stewardship principles [131,132,133]. Within this therapeutic landscape, nitric oxide-releasing gels (NORGs) represent an emerging platform with mechanistic and functional overlap with these established agents but also possess unique biological attributes. A comparative evaluation elucidates both the promise and the current limitations of NORGs relative to other advanced wound dressings.

Silver-based dressings—particularly nanocrystalline and ionic silver formulations—are among the most widely used advanced antimicrobials. Silver exerts broad-spectrum activity through disruption of bacterial membranes, inhibition of respiratory-chain enzymes, and interference with DNA replication [131]. Its clinical utility spans acute surgical wounds, burns, and chronic ulcers. However, silver efficacy is highly dependent on ion-release kinetics, wound exudate composition, and dressing design, and cytotoxicity toward keratinocytes and fibroblasts has been reported at sustained high concentrations [134]. Concerns about silver tolerance and emerging resistance in wound isolates have been increasingly documented, prompting stewardship-driven recommendations for short, targeted use limited to periods of high bioburden or local infection [135]. These considerations have motivated interest in adjunctive or alternative non-antibiotic agents capable of similar broad-spectrum effects with lower cytotoxic and stewardship concerns.

Iodine-based dressings, including povidone-iodine and cadexomer iodine, offer potent activity against bacteria, fungi, and certain viruses, and exhibit robust antibiofilm action [136]. Modern iodine formulations have reduced cytotoxicity compared with older preparations, and their utility is particularly evident in sloughy or heavily exudative chronic wounds. Although thyroid dysfunction is rare when used appropriately, caution is warranted in large-surface-area wounds or patients with thyroid disease [136]. Iodine remains an essential and widely validated antiseptic within chronic wound care, but prolonged use can irritate periwound skin, and its efficacy may be reduced in wounds with dense biofilm or high organic load.

PHMB-impregnated dressings represent a distinct class of non-antibiotic antimicrobials. PHMB, a synthetic cationic polymer structurally analogous to endogenous antimicrobial peptides, binds to bacterial membranes and induces lethal permeability changes. These dressings have demonstrated broad-spectrum antimicrobial activity, good tolerability, and comparatively low cost [137]. Clinical evidence suggests that PHMB dressings can reduce bacterial burden and may improve healing trajectories in chronic wounds, although large randomized controlled trials remain limited [138]. In in vitro biofilm models, PHMB often performs favorably, though outcomes vary depending on the specific model and microbial species involved [139]. PHMB’s relatively low propensity for resistance development and its compatibility with AMS frameworks make it a pragmatic choice for many wound-care settings.

Octenidine dihydrochloride, widely used in Europe, has gained prominence as a cost-effective, broad-spectrum topical antiseptic for colonized and locally infected wounds. Its mechanisms include membrane disruption, interference with cell-wall synthesis, and inhibition of microbial metabolism. Octenidine exhibits strong antibiofilm activity and favorable tolerability profiles in comparative studies [140]. Although clinical evidence consists largely of smaller trials, case series, and regional guidelines, its incorporation into local AMS strategies continues to expand, reflecting its value in reducing reliance on topical antibiotics [140,141].

Medical-grade honey (MGH) dressings, derived from standardized honey preparations such as Manuka honey, exert antimicrobial effects through high osmolarity, hydrogen peroxide production, low pH, and phytochemical constituents. Clinical trials and observational studies have shown that MGH can assist debridement, reduce infection, and accelerate healing in certain acute and chronic wounds [101]. Nonetheless, heterogeneity among honey types, formulations, and comparator treatments complicates meta-analytic conclusions, and evidence quality across studies is moderate to low [102]. Despite these limitations, MGH is widely regarded as a safe, cost-effective, antibiotic-sparing modality that fits well within stewardship frameworks [102].

When compared with these established non-antibiotic agents, nitric oxide-releasing gels exhibit several overlapping and several unique mechanistic features. Like silver, iodine, PHMB, and octenidine, NORGs provide broad-spectrum antimicrobial activity, including efficacy against multidrug-resistant organisms such as MRSA, vancomycin-resistant enterococci, carbapenem-resistant *Enterobacterales*, and *Pseudomonas aeruginosa* [117,118,119]. Unlike many antiseptics, however, NORGs exert antimicrobial effects through multi-target oxidative and nitrosative mechanisms that simultaneously disrupt DNA, proteins, membranes, and metabolic enzymes, thereby minimizing resistance potential [101,102,103,104]. These multitiered effects position NORGs conceptually alongside the broad-spectrum antiseptics central to modern AMS strategies [131,132,133].

NORGs also exhibit potent antibiofilm effects comparable to or exceeding those of silver, iodine, PHMB, and octenidine in several in vitro and animal studies. Nitric oxide disrupts biofilms by modulating cyclic-di-GMP signaling, triggering dispersal of sessile bacteria into planktonic forms that are more susceptible to host defenses and adjunctive treatments [120]. This biofilm-disruptive capacity addresses one of the most significant limitations of classical antibiotics and enhances the theoretical value of NORGs in chronic wound management. While silver, iodine, PHMB, and octenidine also possess antibiofilm activity, their efficacy varies by microbial species, wound environment, and formulation, whereas NO’s signaling-mediated biofilm dispersal mechanism appears more consistent across taxa [134,135,136,137,138,139,140].

A distinguishing advantage of NORGs is their intrinsic pro-healing activity—a property not shared by most other non-antibiotic wound dressings. Nitric oxide promotes angiogenesis, enhances fibroblast proliferation and collagen synthesis, modulates inflammation, and improves microvascular perfusion through activation of the sGC–cGMP–PKG pathway [88,92,107]. These host-directed effects directly target the cellular and microenvironmental abnormalities characteristic of chronic wounds, including impaired vascularization, fibroblast senescence, persistent inflammation, and poor epithelial migration [89,90,91,92]. In comparison, silver, iodine, PHMB, and octenidine do not directly stimulate tissue repair and may exhibit cytotoxicity toward keratinocytes or fibroblasts at high concentrations [134,135,136,138].

From a safety perspective, NORGs demonstrate favorable profiles in early human studies, with minimal systemic absorption and low rates of local irritation. Importantly, unlike neomycin and bacitracin, NORGs have shown no evidence of allergic sensitization in available studies, potentially reflecting the endogenous nature of NO and the biocompatibility of hydrogel matrices [21,22,23,122]. In contrast, silver and iodine dressings may cause cytotoxicity, and honey-based dressings occasionally trigger stinging or allergic reactions [101,102,134,135,136]. However, long-term safety data for NORGs remain limited, and larger clinical trials are required to confirm tolerability across diverse wound etiologies and patient populations.

Despite their substantial promise, NORGs do not yet match the depth of clinical evidence supporting silver, iodine, PHMB, octenidine, and MGH. While multiple preclinical studies and early-phase human investigations demonstrate strong antimicrobial and pro-healing effects, high-quality randomized controlled trials comparing NORGs directly with established non-antibiotic dressings are lacking [117,118,119,120,121,122,123]. As a result, NORGs should presently be viewed as emerging additions to the non-antibiotic antimicrobial armamentarium rather than as established alternatives. Their potential alignment with stewardship principles is compelling, but formal placement within AMS-driven care pathways awaits robust comparative effectiveness data [88,92,131,132,133,134,135,136,137,138,139,140,141,142,143].

When viewed in the aggregate, NORGs share key antimicrobial and antibiofilm features with advanced non-antibiotic wound dressings while uniquely contributing pro-reparative biological activity. This places them at a conceptual intersection between antimicrobial therapy and regenerative wound care—a dual functionality unmet by existing topical agents. However, their optimal clinical role will depend on forthcoming evidence clarifying comparative efficacy, safety, cost-effectiveness, and performance across varied wound types and microbial burdens.

### 3.7. Clinical Evidence, Safety, Implementation Considerations, and Future Directions for NORGs

The clinical evidence supporting nitric oxide-releasing gels (NORGs) has expanded substantially over the past two decades, progressing from foundational in vitro studies to animal models and early-phase investigations in humans. Although still emerging compared with established antiseptic dressings such as silver, iodine, polyhexamethylene biguanide (PHMB), octenidine, and medical-grade honey, the translational trajectory of NORGs provides strong mechanistic and biological rationale for their future integration into wound-care practice [89,90,91]. This section synthesizes the current state of clinical evidence, evaluates safety profiles, and considers practical implementation challenges that must be addressed to ensure successful incorporation of NORGs into antimicrobial stewardship (AMS) frameworks.

### 3.8. Early Clinical Trials and Case Studies

Human clinical evaluation of NORGs remains limited but promising. Early first-in-human studies assessing topical NO-releasing preparations demonstrated rapid reduction in bacterial burden and favorable effects on wound closure trajectories. In a Phase 1 trial evaluating a NO-releasing ointment for chronic leg ulcers, treatment was associated with improved rates of re-epithelialization, reductions in wound size, and a trend toward enhanced granulation tissue quality, with no significant systemic absorption of NO metabolites detected [122]. Local tolerability was excellent, and adverse effects were limited to transient mild irritation.

Another pilot investigation examining a sustained-release NO nanoparticle formulation in patients with chronic wounds reported reductions in Staphylococcus aureus colonization, enhanced granulation tissue formation, and improved wound-bed quality [142]. Similar findings were observed in a study using NO-releasing hydrogels to treat diabetic foot wounds, in which enhanced perfusion, accelerated epithelial migration, and significant reductions in inflammatory biomarkers were documented [143]. Although these studies were small and uncontrolled, they provide early clinical evidence consistent with the robust results seen in preclinical models.

Additional small-scale evaluations have demonstrated benefit in acute wounds. In a clinical series involving postsurgical incisions and traumatic lacerations, NO-releasing topical formulations were associated with reduced erythema, faster wound maturation, and minimal scarring compared with inert gel comparators [144]. These findings align with NO’s well-established roles in modulating inflammation, improving microvascular perfusion, and supporting extracellular matrix remodeling.

While these studies support the translational potential of NORGs, larger controlled clinical trials are required to determine comparative efficacy, long-term safety, and their place in clinical guidelines.

### 3.9. Antimicrobial Outcomes in Human Studies

The antimicrobial performance of NORGs in clinical settings mirrors findings from in vitro and in vivo studies. Across early trials, NO-releasing preparations have consistently reduced microbial load, including colonization by MRSA, *Pseudomonas aeruginosa*, and mixed-species biofilms common in chronic wounds [122,142,143,144]. Importantly, reductions in bacterial burden were accompanied by improved wound-healing parameters, suggesting that NO’s dual antimicrobial and pro-regenerative effects may address both microbial and host dysfunctions underlying chronic wound pathology.

Although direct comparisons with silver, iodine, or PHMB dressings have not yet been conducted in randomized controlled trials, the preliminary clinical data support the hypothesis that NORGs can achieve levels of antimicrobial reduction comparable to established antiseptics while providing additional pro-healing advantages. This dual action distinguishes NORGs from most topical antimicrobials and underscores their potential as multifunctional agents appropriate for AMS-aligned wound care.

### 3.10. Safety Profile and Tolerability

Safety considerations for NORGs focus primarily on local tolerability, potential systemic absorption, oxidative tissue injury, and sensitization. Across available human studies, NORGs have demonstrated excellent safety profiles. Systemic NO exposure, as measured by plasma nitrite, nitrate, and methemoglobin levels, has remained well within normal physiological ranges. No significant changes in heart rate, blood pressure, or oxygen saturation have been observed during or after treatment [122,144]. This is consistent with NO’s rapid local metabolism and the controlled-release kinetics provided by hydrogel matrices.

Local adverse effects are rare and typically mild. Transient stinging or burning sensations have occasionally been reported but usually resolve spontaneously or with adjustment of application frequency [122,142]. Importantly, there have been no documented cases of allergic contact dermatitis attributable to NORGs. This contrasts sharply with neomycin and bacitracin, which exhibit high sensitization rates and are leading causes of medication-induced allergic dermatitis [21,22,23]. The absence of sensitization with NORGs likely reflects the endogenous nature of NO and the inert composition of most hydrogel vehicles.

Potential concerns surrounding oxidative tissue injury at high NO concentrations appear negligible at therapeutic fluxes delivered by NORGs. While NO can combine with superoxide to form peroxynitrite, a potent oxidant, NORG formulations release NO at controlled rates that mimic physiological levels, avoiding the cytotoxic thresholds observed in some inhaled or systemic NO therapies [101,102,103,104,122]. Nevertheless, long-term safety in diverse patient populations—including those with large wounds, extensive comorbidities, or severe peripheral arterial disease—requires further systematic evaluation.

### 3.11. Challenges in Manufacturing, Stability, and Standardization

Despite encouraging biological and clinical properties, several challenges must be addressed before NORGs can be integrated into routine clinical practice. Manufacturing stability represents a key hurdle. Many NO donors degrade over time or under certain storage conditions, reducing NO release capacity and compromising therapeutic efficacy [108,123]. Advances in donor encapsulation, xerogel stabilization, and controlled-humidity packaging have mitigated these challenges but have not eliminated them entirely.

Equally important is the need for standardized NO flux measurements. NO release depends on donor chemistry, hydrogel thickness, hydration status, wound exudate composition, temperature, and pH [108,123]. Without standardized methods to quantify bioavailable NO, comparing different formulations or ensuring consistent therapeutic delivery remains challenging. Regulatory pathways will require validated, reproducible methods for characterizing NO flux before granting approval for widespread clinical use.

Another practical consideration is cost. Manufacturing NO donors and hydrogel matrices at commercial scale may be more expensive than producing conventional antiseptic dressings [123]. While economies of scale may reduce costs over time, initial pricing could limit early adoption, especially in resource-constrained healthcare settings. However, the potential for NORGs to accelerate healing, reduce need for systemic antibiotics, and decrease overall wound-care resource utilization may ultimately justify higher upfront costs.

At present, several inhaled nitric oxide (NO) products are FDA-approved for indications such as neonatal pulmonary hypertension, demonstrating longstanding regulatory familiarity with NO as a therapeutic agent. More recently, the FDA approved berdazimer 10.3% gel, a topical nitric oxide-releasing medication for the treatment of molluscum contagiosum, marking the first regulatory authorization of a topical NO-releasing drug product in the United States. This approval establishes an important regulatory precedent for nitric oxide–based topical therapies and confirms the feasibility of safely delivering exogenous NO through dermatologic formulations.

However, aside from berdazimer—which is indicated specifically for a viral infection—topical NO-releasing gels intended for antimicrobial or wound-healing applications remain under active clinical investigation and have not yet achieved broad regulatory approval for wound-care indications. Some nitric oxide–based dressings and related devices have received CE-mark certification in Europe under device frameworks, but these are distinct from pharmaceutical approvals.

Thus, while NO is now an FDA-approved therapeutic modality in both inhaled and topical forms, NORGs developed for bacterial infections or wound-healing indications remain in clinical development, and additional high-quality clinical evidence will be required before regulatory submissions for wound-care applications can be advanced and approved.

### 3.12. Integration into Antimicrobial Stewardship Frameworks

NORGs align conceptually with AMS principles by offering multi-target antimicrobial activity, low resistance risk, antibiofilm effects, and intrinsic pro-healing properties as summarized in Table 3. Their potential to reduce reliance on topical antibiotics and, in some cases, systemic antimicrobials underscores their future value in AMS-guided care [101,102,131,132,133,134,135,136,137,138,139,140,141]. However, integration into stewardship guidelines will require clear evidence regarding:

1. Comparative antimicrobial efficacy against silver, iodine, PHMB, and octenidine;
2. Performance in biofilm-rich chronic wounds;
3. Long-term safety across diverse patient populations;
4. Cost-effectiveness and real-world clinical impact;
5. Optimal indications and contraindications in wound-care pathways;
6. Until large, multicenter randomized controlled trials are completed, NORGs should be viewed as promising investigational agents with strong mechanistic justification but limited definitive clinical evidence.

## 4. Future Directions and Research Priorities

Key research priorities include:

Large randomized controlled trials: Rigorous head-to-head comparisons of NORGs versus silver, iodine, PHMB, and octenidine in chronic wounds are essential to determine relative efficacy and stewardship value [145].

Expanded mechanistic studies: Additional exploration of NO’s effects on fibroblast senescence, macrophage polarization, diabetic microvascular dysfunction, and redox biology will refine therapeutic indications [146].

Optimization of delivery systems: Advances in hydrogel rheology, donor chemistry, and responsive materials (e.g., pH-triggered or biofilm-sensing release) may enhance efficacy and safety [147].

Pharmacoeconomic analyses: Evaluating the impact of NORGs on healing rates, clinic visits, antibiotic prescribing, and overall healthcare resource utilization will be indispensable for policy and guideline adoption [148].

Real-world implementation research: Registries and surveillance programs capturing long-term safety, resistance trends, and practical use patterns will help define optimal clinical integration [149].

## 5. Discussion and Conclusions

The evolving understanding of wound biology, antimicrobial resistance, and biofilm recalcitrance has fundamentally reshaped the therapeutic landscape of topical wound management. Classical topical antibiotics—once widely regarded as benign, convenient, and broadly effective—are now recognized as having significant limitations that restrict their utility within modern antimicrobial stewardship (AMS) frameworks. Rising resistance to mupirocin, fusidic acid, and neomycin, coupled with high rates of allergic contact dermatitis and poor penetration into biofilm-dominated chronic wounds, underscores the inadequacy of these agents for many contemporary wound-care challenges. Their narrow antimicrobial spectra and absence of pro-healing properties further diminish their role in treating the complex microenvironmental disturbances that characterize chronic wounds.

In contrast, non-antibiotic topical antimicrobials such as silver, iodine, polyhexamethylene biguanide (PHMB), octenidine, and medical-grade honey have emerged as preferred alternatives, offering broad-spectrum activity with relatively low risks of resistance development. These agents align more closely with the multifactorial nature of chronic wound pathophysiology and have become integral components of AMS-guided topical therapy [101,102,131,132,133,137,138,139,140,141]. However, despite their broad antimicrobial activity and antibiofilm potential, these modalities primarily target microbial bioburden and provide limited direct support for the impaired host processes that prevent wound healing.

Nitric oxide-releasing gels (NORGs) represent a notable departure from both classical antibiotics and established non-antibiotic antimicrobials by combining multi-target antimicrobial activity with host-directed pro-healing effects. Mechanistically, NO disrupts microbial membranes, respiratory enzymes, protein synthesis, and DNA integrity through oxidative and nitrosative reactions that are not easily circumvented by single-gene mutations, thereby conferring an inherently low risk of resistance [101,102,103,104,150]. NO’s ability to modulate quorum-sensing pathways and trigger biofilm dispersal directly addresses one of the principal barriers to effective chronic wound treatment. These antimicrobial properties are complemented by NO’s central physiological roles in angiogenesis, immune regulation, fibroblast activation, keratinocyte migration, extracellular matrix synthesis, and microvascular perfusion [88,92,96,97,98,99,100,101,102,103,104,105,106,107]. Thus, NORGs provide a multifunctional therapeutic platform capable of addressing both microbial burden and the underlying biological impairments characteristic of chronic wounds.

The clinical evidence supporting NORGs, while still emerging, offers compelling proof of concept. Early-phase trials and case studies consistently demonstrate reductions in bacterial burden, improvements in granulation tissue quality, accelerated re-epithelialization, and favorable tolerability profiles, with minimal systemic absorption and no documented cases of allergic sensitization [122,142,143,144]. These outcomes align closely with the mechanistic rationale for NO therapy and suggest substantial translational potential. Nevertheless, the current body of evidence remains insufficient to define optimal indications or to establish NORGs as first-line agents in AMS-driven wound-care pathways. Large randomized controlled trials comparing NORGs to established antiseptic dressings are urgently needed to provide definitive data on comparative effectiveness, safety, and cost-related implications [145,146,147,148,149].

A critical dimension of AMS is the recognition that topical agents must be deployed judiciously, guided by clinical signs of infection rather than colonization alone. This principle applies equally to NORGs. While NO’s broad-spectrum antimicrobial effects and low resistance potential are attractive, indiscriminate use would conflict with stewardship goals and risk unnecessary cost and treatment exposure. Future clinical guidelines will need to delineate clear criteria for NORG deployment, integrating microbial assessments, wound characteristics, host factors, and broader stewardship considerations.

From a mechanistic standpoint, NO’s capacity to directly address key barriers to healing—including microvascular dysfunction, excessive inflammation, fibroblast senescence, impaired collagen synthesis, and biofilm persistence—positions NORGs at a conceptual intersection between antimicrobial therapy and regenerative medicine. This duality suggests that NORGs may be particularly beneficial for chronic wounds that exhibit a combination of moderate infection, high bioburden, or biofilm presence alongside impaired tissue repair. However, detailed understanding of dose–response relationships, optimal treatment regimens, and potential synergy or interaction with established wound therapies will be essential for maximizing therapeutic benefit.

Future research must address several key areas. Comparative effectiveness trials are needed to evaluate the performance of NORGs against silver, iodine, PHMB, octenidine, and honey across diverse wound etiologies. Mechanistic studies should further elucidate NO’s effects on macrophage polarization, fibroblast senescence reversal, and redox biology in chronic wound environments. Pharmacoeconomic analyses will determine whether NORGs reduce overall healthcare burden by accelerating healing, reducing clinic visits, or decreasing systemic antibiotic use. Long-term safety studies, including in patients with extensive comorbidities or large wound surface areas, will clarify whether chronic or recurrent NO exposure poses any risks. Development of standardized NO flux measurement protocols will also be crucial for regulatory approval and clinical adoption [108,123,124,148].

In conclusion, classical topical antibiotics increasingly fail to meet the demands of modern wound care due to limited antimicrobial coverage, high resistance potential, allergenicity, and lack of host-directed benefits. Advanced non-antibiotic wound dressings offer improved stewardship profiles but remain primarily antimicrobial in action. Nitric oxide-releasing gels occupy a unique therapeutic niche by combining broad-spectrum antimicrobial and antibiofilm activity with biologically meaningful enhancement of angiogenesis, inflammatory regulation, extracellular matrix deposition, and microvascular perfusion. These properties hold promise for addressing the complex, multifactorial pathophysiology of chronic wounds in ways that neither conventional antibiotics nor antiseptics can fully achieve.

Furthermore, NORGs may help mitigate antibiotic resistance by providing a non-traditional antimicrobial modality that bypasses the canonical targets exploited by conventional antibiotics. Because NO targets multiple microbial structures and processes simultaneously, it avoids the genetic bottlenecks that enable rapid resistance selection. Additionally, the ability of NO to disperse and eradicate biofilms may reduce the need for prolonged or repeated antibiotic courses, thereby decreasing selection pressure in chronic wounds.

However, until robust clinical evidence accumulates, NORGs should be regarded as promising investigational technologies rather than replacements for established therapies. Their ultimate role in wound care will depend on continued scientific validation, regulatory refinement, comparative clinical trials, and integration into stewardship-aligned practice guidelines. As research progresses, NORGs may emerge as a cornerstone of next-generation topical wound therapy—bridging the longstanding gap between antimicrobial efficacy and regenerative healing in chronic and complex wounds.

## Figures and Tables

**Table 1:** Resistance Mechanisms and Prevalence for Common Topical Antibiotics.

Antibiotic	Major Resistance Mechanisms	Genetic Determinants	Approximate Prevalence
Mupirocin	Low-level: point mutations in *ileS*; High-level: acquisition of alternate isoleucyl-tRNA synthetase	*mupA*, *mupB*	Up to 30% in some MRSA-endemic hospitals
Fusidic Acid	EF-G mutations; plasmid-mediated resistance	*fusA*, *fusB*, *fusC*, *fusD*	>40% in some regions with high impetigo burden
Neomycin	Aminoglycoside-modifying enzymes, efflux pumps, rRNA methylation	*aac*, *aph*, *ant*, *armA*	Increasing globally; often cross-resistant with systemic AGs
Polymyxin B	Modification of lipid A	*mcr-1* to *mcr-10*	Low but rising globally
Bacitracin	Overexpression of efflux and resistance operons	*bcrABC*	Variable, increasing in chronic wound isolates

**Table 2:** Functional Roles of Nitric Oxide in Wound Biology.

Function	Mechanistic Basis	Effects in Wounds
Antimicrobial Activity	Nitrosative/oxidative stress; DNA damage; enzyme inhibition	Kills bacteria, fungi, and select viruses; active vs. MDROs
Biofilm Disruption	NO-mediated reduction of c-di-GMP	Promotes dispersal and eradication
Angiogenesis Support	sGC–cGMP pathway; endothelial migration	Increased perfusion, granulation
Inflammation Modulation	Macrophage polarization; cytokine reduction	Resolves chronic inflammation
Fibroblast Activation	NO-mediated proliferative signaling	Enhanced collagen deposition
Re-epithelialization	Keratinocyte migration	Accelerated closure

**Table 3:** Comparative Evaluation: Traditional Antibiotics vs. Antiseptics vs. NORGs.

Feature	Topical Antibiotics	Non-Antibiotic Antiseptics (Silver/Iodine/PHMB/Octenidine/Honey)	NORGs
Spectrum	Narrow	Broad	Broad
Biofilm Efficacy	Poor	Moderate–high	High
Resistance Risk	High	Low	Very low
Allergic Potential	High (bacitracin, neomycin)	Low	Very low
Healing Modulation	None	Limited	Strong
Activity vs. MDROs	Poor	Moderate	Strong
Stewardship Alignment	Weak	Strong	Potentially strong
Evidence Base	Extensive but declining utility	Strong	Emerging

## Data Availability

Data is contained within the article.
